# In depth evaluation of the prognostic and predictive utility of PTEN immunohistochemistry in colorectal carcinomas: performance of three antibodies with emphasis on intracellular and intratumoral heterogeneity

**DOI:** 10.1186/s13000-016-0508-0

**Published:** 2016-07-08

**Authors:** Emese Irma Ágoston, Tamás Micsik, Balázs Ács, Krisztina Fekete, Oszkár Hahn, Zsolt Baranyai, Kristóf Dede, György Bodoky, Attila Bursics, Janina Kulka, Tibor Krenács, Balázs Győrffy, László Harsányi, A. Marcell Szász

**Affiliations:** Department of Surgery, Semmelweis University, 78 Üllői út, Budapest, 1082 Hungary; Department of Pathology and Experimental Cancer Research, Semmelweis University, 26 Üllői út, Budapest, 1085 Hungary; Department of Pathology, Semmelweis University, 93 Üllői út, Budapest, 1091 Hungary; Department of Surgery and Oncological Surgery, Uzsoki Teaching Hospital, 196 Róna utca, Budapest, 1145 Hungary; Department of Oncology, Szent István Hospital, 1 Nagyvárad tér, Budapest, 1097 Hungary; MTA-TTK Lendület Cancer Biomarker Research Group, Magyar tudósok körútja 2, Budapest, 1117 Hungary

**Keywords:** Colorectal cancer, PTEN, Protein expression, Intratumoral heterogeneity, Prognostic marker, Predictive marker, Immunohistochemistry

## Abstract

**Background:**

Phosphatase and tensin homolog deleted in chromosome 10 (PTEN) loss of function is frequently detected in advanced colorectal cancer. Its detection is thought to have prognostic significance and it is being considered to predict responsiveness to anti-EGFR therapy. Unfortunately, while immunohistochemical assessment of PTEN expression is widespread, it lacks standardization and the results are hardly comparable across the available publications.

**Methods:**

Retrospectively collected, formalin-fixed and paraffin-embedded colorectal tumor tissue samples from 55 patients were combined into tissue microarray (TMA) blocks. We used three different PTEN antibodies to determine the frequency, intensity and intracellular pattern of PTEN immunohistochemical labeling: Neomarkers, Dako and CellSignaling. We evaluated the aforementioned parameters in selected regions of colorectal cancers and in their lymph node metastases by using three scoring methods that take into consideration both staining frequency and intensity (H1-H3-score). We also evaluated intracellular localization.

**Results:**

The Dako and CellSignaling antibodies stained predominantly cytoplasms, while the Neomarkers antibody specifically stained cell nuclei. PTEN H-scores were significantly lower in all tumor areas as compared to the normal colonic mucosa based on staining with the DAKO and CellSignaling antibodies. Intratumoral regional differences or differences between matching tumors and metastases were not detected with any of the antibodies. Neither Dako, neither CellSignaling, nor the Neomarkers antibodies revealed a significant correlation between PTEN expression and pT, Dukes/MAC and clinical stage. KRAS status, histological grade correlated with PTEN H-scores based on staining with the Neomarkers antibody. PTEN H-scores did not correlate with MMR status. PTEN H-scores did not show any correlation with relapse-free survival based on staining with either antibody.

**Conclusions:**

While PTEN expression decreased in colorectal cancer according to two antibodies, neither of the three applied PTEN antibodies could justify significant correlation with clinicopathological data, nor had prognostic value. Thus, we might conclude that immunohistochemical PTEN investigation remains a challenge requiring more standardized evaluation on larger number of cases to clarify its utility as a prognostic and predictive tool in CRC. The standardization of immunohistochemical method is key in the evaluation process, which is further discussed.

**Electronic supplementary material:**

The online version of this article (doi:10.1186/s13000-016-0508-0) contains supplementary material, which is available to authorized users.

## Background

Colorectal cancer (CRC) is the third most commonly diagnosed malignant disease in men and second in women. Its incidence is 1.2 million cases per year, and 608.700 deaths are estimated to have occurred in year 2008 all over the world [1]. So far, prognostic markers are limited to clinicopathological properties of colorectal tumors (TNM7 and Dukes/modified by Astler-Coller/stage classifications, tumor budding and lymphovascular and perineural invasion). Microsatellite instability (MSI) is an established predictive marker and determination of *KRAS* status is important in the metastatic setting. Recently, mutations of *BRAF*, *PTEN*, *PIK3CA* and other *RAS* genes have been identified to be predictive for response to anti-EGFR therapy [2, 3].

In the 1990s, two independent studies led to the identification of a tumor suppressor gene *PTEN* (tumor suppressor Phosphatase and TENsin homologue deleted in chromosome 10) in the chromosomal region 10q23. The *PTEN* encodes a protein consisting of 403 amino acids with double phosphatase specificity for both lipid and protein substrates. PTEN is expressed both in the cytoplasm and in the nucleus, playing different roles at the two sites. In the cytoplasm, PTEN is mainly involved in the homeostatic maintenance of *PI3K/AKT* signaling by counteracting the activity of PI3Ks. Thus PTEN regulates cell growth directly by inhibiting cell cycle progression, facilitating cell death, modulating growth arrest signals, and indirectly by limiting angiogenesis. Whereas in the nucleus it is involved in genomic stability and cell cycle regulation [4]. Its loss of function leads to an upregulation of the PI3K/AKT signaling pathway, which in turn triggers cell proliferation and inhibition of apoptosis [5].

Inactivation of PTEN is frequent in multiple cancer types and it can occur through various genetic alterations: loss of heterozygosity (LOH), promoter hypermethylation, point mutations or large chromosome deletions [6–9]. PTEN inactivation has been reported approximately in 20-40 % of CRCs [10, 11]. Mutations seem to be responsible for PTEN-loss in approximately 5-14 % of cases [10–12]. In a recent study, PTEN protein expression was found to be lost approximately in 37 % of CRC without liver metastasis and in 75 % with metastatic tumor. Additionally, in these cases no PTEN protein expression was observed in liver metastases [12]. Multiple reports claimed that intranuclear expression is more important in tumor progression and those studies have found prognostic value for nuclear PTEN loss [13–16]. Another study revealed that PTEN protein localizes to the cytoplasm of both normal and tumor cells and no correlation of immunostaining and tumor characteristics could be confirmed [17]. Thus, the role of PTEN as a prognostic factor in CRC is still controversial [18].

Recently, various studies analyzed the predictive role of PTEN in targeted therapies. Maintained PTEN expression in the tumor tissue appeared to be correlated with response to treatment with the anti-EGFR antibody cetuximab in independent observations [19–22]. Conversely, others found no correlation between PTEN loss and response to anti-EGFR antibodies [17, 23].

There are several methods available for assessing PTEN loss, such as Immunohistochemistry (IHC), FISH, direct sequencing, western-blot, etc. Among these, IHC is the most widespread since it shows lack of PTEN expression independently of its cause in an easy, fast and cost effective way [21, 24, 25]. More studies validated the correlation between PTEN-deletions/mutations and decrease of protein levels detected by IHC [24, 26–28]. On the other hand, evaluation of PTENIHC has not been sufficiently standardized, neither in the (pre-) analytical nor in interpretation/evaluation phase. Different methods such as positivity with any kind of staining vs. no reaction [21, 29], histoscore [23], different cut-off levels for PTEN loss (10 %, 50 %), or similar techniques [22, 30, 31] have been reported. In a recent study, a robust dichotomized immunohistochemical assessment of PTEN expression was performed. A high concordance of PTEN expression levels in matched primary and metastatic CRCs was found and these expression levels significantly correlated with survival [27, 28].

**Fig. 1:**
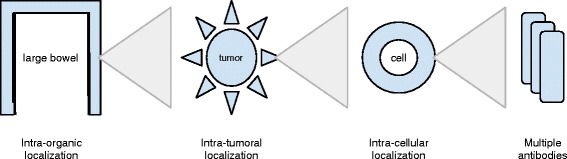
Infographics displaying the aims and methods utilized in the evaluation of PTEN expression in colon carcinoma. We have systematically considered location in the colorectum, intratumoral localization, intracellular staining pattern using three different antibodies

Although PTEN function might play a crucial role in colorectal cancer pathogenesis and progression, the details remain obscure. We still have to understand the significance of localization (nuclear vs. cytoplasmic), to determine appropriate threshold or cut-off values for loss of expression or other ways of meaningful interpretation of IHC . Furthermore, the many commercially available antibodies might have differential specificity and sensitivity. In our study, we aimed at addressing technical, pathobiological and clinically relevant questions by comparative analysis of PTEN-expression in CRC. We analyzed intracellular, intratumoral and locoregional heterogeneity using three different antibodies and assessed the prognostic power of those for distant metastasis-free and overall survival (Fig. 1).

## Methods

Fifty-five patients were included in the study (Table 1), which was approved by the local Institutional Review Board (IKEB #207/2011). The mean age (± standard deviation) was 63.45 ± 9.9 years. Upon enrollment, 28/55 (51 %) and 27/55 (49 %) patients were males and females, respectively. Only tumors with known KRAS status were included in the study: intentionally fifty-one percent of the patients had wild-type and 49 % had mutated KRAS status. Formalin-fixed, paraffin-embedded (FFPE) samples were used. The following areas were selected from all resection specimens: Normal colon, border between intact mucosa and tumor, main tumor mass, invasive front, lymph node metastasis. These regions were re-assessed histologically and marked for core punching using the Tissue Microarray Builder instrument (Histopathology Ltd., Pécs, Hungary). Cores of 2 mm were taken from all sites mentioned above, including duplicates from the regions of main tumor mass and invasive front, where feasible.

**Table 1:** Clinicopathological features of the patients in the evaluation

Variable		Count
Gender	Female	27
	Male	28
Location of tumor	Coecum	9
	Ascend. colon	8
	Hepatic flexure	5
	Transver. Colon	4
	Splenic flexure	4
	Descend. colon	2
	Sigmoid colon	11
	Rectosigmoid	5
	Rectum	7
Grade	NA	8
	Low grade	30
	High Grade	17
pT	NA	11
	2	3
	3	31
	4	10
pN	NA	12
	0	13
	1	15
	2	14
Stage	NA	11
	I	1
	II	13
	III	20
	IV	10
Dukes	NA	11
	A	1
	B	12
	C	21
	D	10
mAC	NA	11
	B1	1
	B2	9
	B3	3
	C1	3
	C2	12
	C3	6
	D	10
MMR (IHC)	NA	12
	MSS	40
	MSI	3
KRAS (RFLP + SEQ)	mut12	24
	mut13	3
	Wt	28

Immunohistochemical analysis of PTEN was performed on 4 μm thick sections cut from TMA blocks mounted on adhesive glass slides (SuperFrost UltraPlus from Gerhard Menzel Ltd., Braunschweig, Germany). After routine dewaxing antigen retrieval was performed either in a pH 6.0 Target Retrieval Solution (Dako, Glostrup, Denmark) for Cell Signaling and Dako antibodies or in a pH 9.0 buffer of 0.01 M Tris-0.1 M EDTA for Neomarkers antibody at ~105 °C for 30 min using an electric pressure cooker (Avair Ida, YDB50-90D, Biatlon Ltd., Pécs, Hungary).

Brief protocol: Endogenous peroxidase activity was blocked in a 0.5 % hydrogen peroxide methanol solution for 20 minutes. Sections were treated in a humidifying chamber at room temperature using the protein block of the Novolink kit for 10 min. Three different, validated [14, 27, 28] and widely used anti-PTEN antibodies were utilized in our study. Two with cytoplasmic staining pattern: PTEN Clone 6H2.1 Monoclonal Mouse (Code M3627, Dako, Glostrup, Denmark) in 1:100 dilution, PTEN 138G6 Rabbit Monoclonal antibody (9559, Cell Signaling, Boston, MA, USA) in 1:40 dilution. And one with a nuclear predominance reactivity: PTEN Ab6 28H6 Monoclonal Mouse (MS1797, Neomarkers, Freemont, CA, USA) in 1:100 dilution. The sections were incubated overnight with primary antibodies diluted in 1 % bovine serum albumin in Tris Buffered Saline (TBS). Subsequently, the slides were treated with the post-primary reagent of the Novolink kit for 30 minutes and then with the Novolink Polymer Detection Systems (Leica-NovoCastra, Newcastle Upon Tyne, UK) kit for an additional 30 minutes. The slides were washed between all incubation steps for 3 min in TBS containing 0.01 % Tween-20. Enzyme activity was visualized using a hydrogen peroxide/DAB (DiAmino Benzidine) solution at pH 4.5 for 3.5 minutes, internal controls (neural elements and endothel) were utilized for each reaction. Finally, the slides were counterstained with hematoxylin.

Digital imaging of immunostained specimens was performed using a Pannoramic P250beta slide scanner (3DHistech Ltd., Budapest, Hungary). PTEN expression patterns were analyzed using the TMA module of the 3DHistech Pannoramic Viewer (1.15.2 RTM) using the following dimensions: intracellular localization (nuclear, cytoplasmic, nuclear and cytoplasmic); intensity (0: none, 1: weak, 2: intermediate, 3: strong expression); proportion (0: none, 1: 0-1 %, 2: 2-10 %, 3: 11-33 %, 4: 34-66 %, 5: 67-100 % of respective cells stained). Scores from the duplicate areas were averaged and raw data was used for statistical evaluation. For statistical analyses the H-score for a tumor region was calculated with three different scoring systems: a) multiplying intensity (i) with frequency (f) resulting in a 0–15 range (H1-score_tumor region_ = i_tumor region_ x f_tumor region_); b) summing intensity (i) and frequency (f) resulting in a 0–8 range (H2-score_tumor region_ = i_tumor region_ + f_tumor region_); c) H3-score weighted towards intensity of staining resulting in a range of 0–15 (Table 2). For prognostic studies all data from respective tumor regions were averaged into a final number representing the staining for a case.

Chi-square test, Friedman test and Wilcoxon test were used for comparison of the expression of PTEN in heterogeneous areas of the tumors and across antibodies. Kruskal-Wallis and Mann–Whitney tests were applied to compare PTEN expression with conventional prognostic markers. ROC (receiver operating characteristic) analysis was used for dichotomizing PTEN expression. Cohen’s kappa was utilized to compare the staining properties. Kaplan-Meier method was applied to display the prognostic value supported by log-rank test. The statistical analysis was performed with SPSS 22 (IBM, Corp., Armonk, NY, USA). All tests were two-sided and *p*-values of less than 0.05 were accepted as statistically significant.

**Table 2:** H3-score. Intersection of corresponding row and column produces H3-score

		Frequency				
	H3-score	0-1 %	1-10 %	10-33 %	33-66 %	67-100 %
Intensity	1+	1	2	3	4	5
	2+	6	7	8	9	10
	3+	11	12	13	14	15

## Results

### Technical comparison of the evaluated antibodies and their intracellular staining pattern

**Fig. 2:**
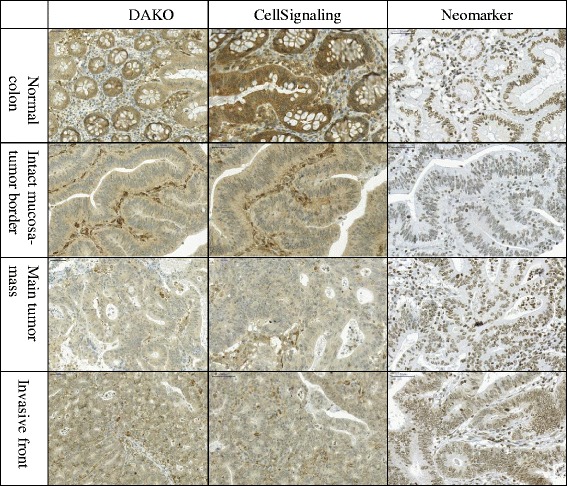
Immunohistochemical images of the investigated regions according to the respective PTEN antibody. The DAKO, CellSignaling and Neomarkers antibodies staining the normal colon, tumor-normal border, main tumor mass and invasive front of the colorectal tumors (20x magnification)

First, we tested the expression of PTEN assessed by the three antibodies in all tumor regions to achieve an overall technical comparison (Fig. 2). The frequency, intensity and location of PTEN expression in all stained cores were evaluated (Table 3). We noted that all antibodies tended to stain the tissues in a fairly homogenous manner regarding frequency of cells once detecting PTEN, however the intensity was lower for the DAKO and CellSignaling antibodies, while higher for the Neomarkers. The latter specifically stained cell nuclei, while the other two antibodies displayed simultaneous nuclear and cytoplasmic staining in most specimens. There was a fair agreement between staining with the Dako and CellSignaling antibodies in frequency, intensity and location, whereas the Neomarkers antibody showed slight agreement with other antibodies.

**Table 3:** Expression of PTEN according to the three antibodies

DAKO				Neomarkers			Cell Signaling		
		Frequency	Valid Percent		Frequency	Valid Percent		Frequency	Valid Percent
Frequency	0	12	5.6	0	2	.8	0	7	3.0
	1 = 0-1 %	13	6.0	1 = 0-1 %	3	1.3	1 = 0-1 %	4	1.7
	2 = 2-10 %	12	5.6	2 = 2-10 %	27	11.4	2 = 2-10 %	2	.8
	3 = 11-33 %	8	3.7	3 = 11-33 %	37	15.6	3 = 11-33 %	5	2.1
	4 = 34-66 %	23	10.6	4 = 34-66 %	27	11.4	4 = 34-66 %	15	6.3
	5 = 67-100 %	148	68.5	5 = 67-100 %	141	59.5	5 = 67-100 %	204	86.1
	Total	216	100.0	Total	237	100.0	Total	237	100.0
Statistics	DAKO vs. NEOM	Value	p	NEOM vs. CELLS	Value	p	CELLS vs. DAKO	Value	p
	Kappa	.067	.066	Kappa	.056	.066	Kappa	.264	.000
	Chi-square		0.064	Chi-square		.286	Chi-square		.000
Intensity	0	12	5.6	0	2	.8	0	7	3.0
	1 = +	158	73.1	1 = +	41	17.3	1 = +	146	61.6
	2 = ++	29	13.4	2 = ++	61	25.7	2 = ++	33	13.9
	3 = +++	17	7.9	3 = +++	133	56.1	3 = +++	51	21.5
	Total	216	100.0	Total	237	100.0	Total	237	100.0
Statistics	DAKO vs. NEOM	Value	p	NEOM vs. CELLS	Value	p	CELLS vs. DAKO	Value	p
	Kappa	.051	.051	Kappa	.082	.014	Kappa	.305	.000
	Chi-square		.024	Chi-square		.260	Chi-square		.000
Location	0	11	5.0	0	2	.8	0	8	3.2
	1 = nucl	5	2.3	1 = nucl	241	99.2	1 = nucl	2	.8
	2 = cytop	44	19.9	2 = cytop	0	.0	2 = cytop	22	8.7
	3 = nucl+cytop	161	72.9	3 = nucl+cytop	0	.0	3 = nucl+cytop	220	87.3
	Total	221	100.0	Total	243	100.0	Total	252	100.0
Statistics	DAKO vs. NEOM	Value	p	NEOM vs. CELLS	Value	p	CELLS vs. DAKO	Value	p
	Kappa	-.001	.712	Kappa	-.005	.000	Kappa	.390	.000
	Chi-square		.480	Chi-square		.000	Chi-square		.000

### Comparison of scoring methods

Next, we calculated H-scores (H1, H2, H3) incorporating the intensity (0–3) and frequency (0–5) values. These scoring systems were strongly correlated (ρ = 0.854-0.948) and no significant difference emerged among them (*p* = 0.228-0.666).

### PTEN expression assessed with emphasis on intratumoral distribution

To assess the overall PTEN protein expression at specific regions we applied all the scoring systems, although, statistical data is displayed only for the H1-score since no significant difference was detected among them.

**Fig. 3:**
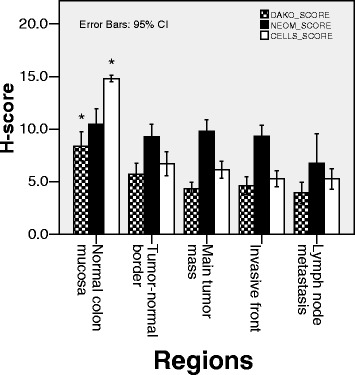
Expression levels of PTEN in normal colon mucosa, in various areas of primary tumor and in metastases. PTEN IHC was performed with antibodies from DAKO, CellSignaling and Neomarkers. Specimens were digitally imaged and H-scores were calculated based on a combination of staining intensity and frequency as described in methods. Data shown are mean PTEN (±95 % confidence interval) H-score values determined by the three antibodies and at the tumor regions indicated. Asterisk (*) marks significant difference (DAKO normal vs. border *p* = 0.005, all others *p* < 0.001 for all others) between the normal colon and all tumor regions detected by the DAKO and the CellSignaling antibody. There were no significant differences based on staining with the Neomarkers antibody

Based on staining with both the DAKO and CellSignaling antibodies, PTEN expression declined from the normal colon mucosa towards tumor progression (tumor-normal border → main mass → invasive front → lymph node metastasis), though, not significantly. PTEN H-scores were significantly lower in all examined tumor areas when compared to the normal colon mucosa (DAKO normal vs. lymph node *p* = 0,109, vs. main mass *p* = 0.005, vs. for all others: border, invasive front and lymph node metastasis *p* < 0.001, respectively; CellSignaling normal vs. all tumor regions *p* < 0.001). Although, displaying a similar trend, the Neomarkers antibody did not detect significant difference between PTEN expression in normal colon and tumor regions (Fig. 3).

There was no significant difference between PTEN expression either between different tumor regions or between matched lymph node metastasis and tumor mass with any of the three antibodies.

### PTEN expression assessed according to tumor localization in the large bowel

**Fig. 4:**
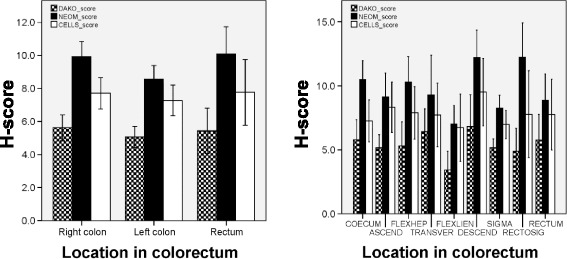
PTEN expression according to the location of the tumor in the large bowel. Mean expression of PTEN (±95 % confidence interval) according to the three antibodies considering location of the tumors in the colorectum

PTEN expression levels assessed by the three antibodies were compared between the various regions of the colorectum (Fig. 4). H-scores of the individual regions (cecum, ascending colon, hepatic flexure, transverse colon, splenic flexure, descending colon, sigmoid colon, rectum) and colon sides (right vs. left vs. rectum) did not show any statistically significant differences in PTEN expression with any of the three antibodies.

### Clinicopathological evaluation

**Fig. 5:**
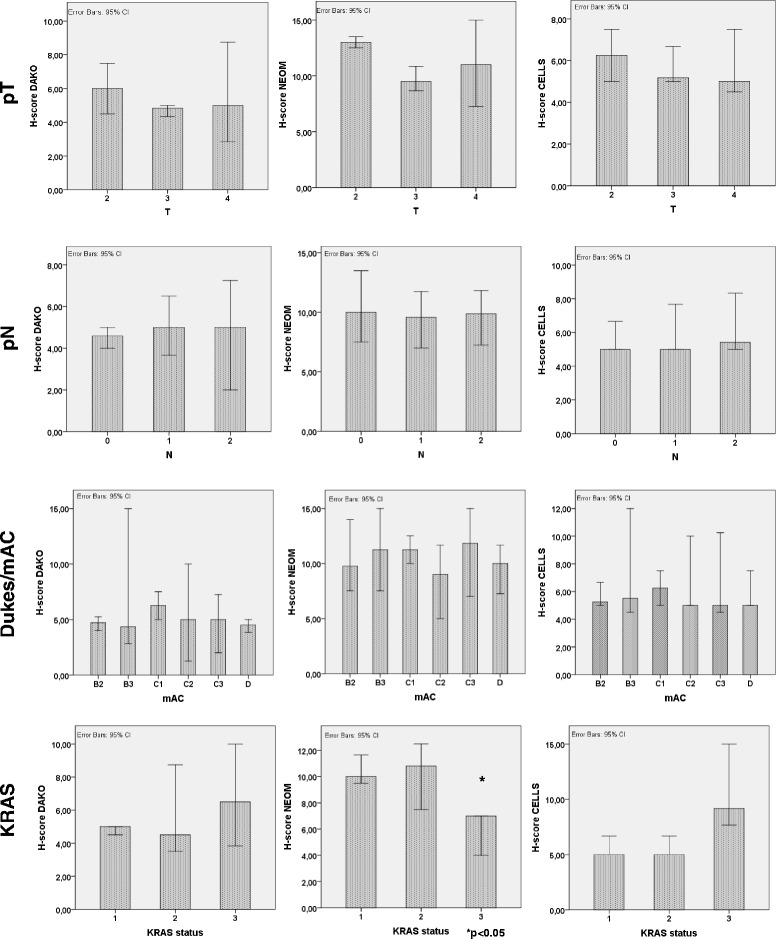
Correlation of PTEN expression and clinicopathological parameters. Mean of PTEN expression investigated by the 3 antibodies according to pT, pN, Dukes-mAC stages and KRAS status

As no significant differences in PTEN protein expression were noted across the selected tumor areas (Fig. 3), we calculated the average score for each case by the following equation: H-score_case_ = (H1-score_tumor-normal border_ + H1-score_main tumor mass_ + H1-score_invasive front_)/3. Next, we analyzed the correlation between the clinicopathological data and the H-scores_case_ (Fig. 5).

Neither DAKO (D), neither CellSignaling (C) nor the Neomarkers (N) antibodies detected any correlation of PTEN expression and pT (p/D/= 0.817, p/N/= 0.175, p/C/= 0.611). Similarly, no significant correlation was noted for Dukes (p/D/= 0.454, p/N/= 0.896, p/C/= 0.824), Dukes-MAC (p/D/= 0.718, p/N/= 0.728, p/C/= 0.990) and for clinical stage either (p/D/= 0.806, p/N/= 0.984, p/C/= 0.727). In contrary, KRAS status correlated with PTEN expression only based on staining by the Neomarkers antibody (p/D/= 0.713, p/N/= 0.029, p/C/= 0.062): PTEN expression detected by the Neomarkers antibody was lower in KRAS mutant tumors bearing the mutation in exon 13. On the other hand, staining by the CellSignaling antibody displayed opposite trend without statistical significance: lower expression in KRASmut at exon 12 and WT tumors as compared to the few KRASmut at exon 13 carcinomas. Similar correlation of grade was seen only with N, showing higher expression in high grade tumors, while equal distribution in all grades was found with D and C (p/D/= 0.832, p/N/= 0.040, p/C/= 0.099). PTEN expression did not correlate with MMR status (p/D/= 0.731, p/N/= 0.315, p/C/= 0.679) based on staining with any of the three antibodies.

For the assessment of prognostic power of PTEN immunohistochemistry we utilized a dichotomizing/binary system on the basis of two methods: A, according to published approaches, maintained PTEN expression (PTEN-normal) was assigned when PTEN expression of the tumor was similar to surrounding normal mucosa [24, 26, 28, 30], whereas PTEN loss was assigned when tumor regions had significantly lower PTEN expression than the surrounding normal mucosa (Additional file 1: Figure S1). B, for a more objective assessment, we have utilized ROC analysis to determine the optimal threshold of PTEN expression detected by immunohistochemistry. Cut-off point of PTEN possesses sensitivity of 61 % and specificity of 70 %, with low area under the curve (AUC: 0.440-0.626). Based on the above-described analyses, no prognostic power of D, C and N antibodies was detected in any setting.

## Discussion

The tumor suppressor gene *PTEN* is gaining attention regarding its role in multiple carcinomas. In CRC, the possible prognostic and predictive value of PTEN immunohistochemical staining is highly investigated, but remains controversial [18, 32, 33].

Whereas more silencing mechanisms (mutations, LOH, promoter hypermethylation, copy number changes or miRNAs) can lead to decreased PTEN-expression or PTEN-loss, one of the most widespread detection method is the immunohistochemistry due to its cost-effectiveness and relative independence from the cause of PTEN loss and the relatively simple procedure. PTEN expression analysis by IHC has been used by the majority of recent studies [18], although, these are not standardized.

Possible differences may occur during the *pre-analytical phase* (such as tissue sampling method, time of ischemia, fixation time, temperature, dehydration conditions) during the *IHC staining process* (antibody concentration, diluent, the different antibody sensitivity and specificity between tissue types, detection method) and during the *interpretation/evaluation phase* (different methods such as positivity with any kind of staining vs. no reaction, histoscore, different cut-off levels for PTEN loss, intracellular localization of PTEN), as well. Considering the results of previous studies, we focused on two key points: antibody type and scoring method. There are more studies validating the different methods assessing PTEN-loss [27, 30], the most valuables are the sequential studies of Sangale et al. They firstly described a thorough validation process of ten different anti-PTEN clones on different cell-lines and samples of known PTEN-status concluding onto the best clones, while in their concomitant study with the ‘winner’ antibody they could demonstrate PTEN’s prognostic role in a set of metastatic CRCs. [27, 28]

We basically followed their approach using their two best ranked antibodies (Dako and CellSignaling) together with on also widely used ‘nuclear’ clone (Neomarkers). As no accepted PTEN-IHC receipt exists now, we used internal controls as suggested by recently mentioned authors.

There are more different scoring methods for PTEN-status with different number of positivity-classes or dichotomization upon the percentage of positive tumor cells compared to internal control. As different studies with different PTEN-classifications and different patient-sets are hardly comparable we decided to use more evaluation methods and to compare the results of those on a common set of cases.

Our study evaluated intracellular and intratumoral heterogeneity of PTEN immunohistochemical detection in CRC using three different antibodies: DAKO (D), Neomarkers (N) and CellSignaling (C). Although different protocols for PTEN immunostaining have been described [27, 30], these are still not standardized. We adopted three combined scoring methods by using three main dimensions: intracellular localization, intensity and frequency of the PTEN expression. To our knowledge, these methods have not been used before. Our goal was to evaluate the staining characteristics with three commercially available antibodies and to correlate those with tumor localization in the colorectum and with clinicopathological data. We attempted to evaluate the prognostic role and clinical applicability of PTEN protein expression in CRC.

There was a moderate correlation between staining with the Dako and the CellSignaling antibodies both in frequency and in intensity, whereas staining with the Neomarkers antibody did not show correlation with other antibodies. The discordant results of N with C and D can be explained partly by the different staining characteristics of the N-antibody favoring nuclear staining, while C and D produced predominantly cytoplasmic staining. Recent validation work by Sangale et al. found that the Neomarkers antibody had low sensitivity and specificity for the detection of PTEN loss [27].

In our study, PTEN expression was significantly lower in all examined tumor areas when compared to the normal colon mucosa by using either DAKO or CellSignaling antibody. The Neomarkers antibody showed similar trend, but without reaching significance. Staining for PTEN levels with all three antibodies exhibited a trend (although without statistical significance) along the tumor edge → main tumor mass → invasive front → lymph node metastasis sequence, which is in agreement with a potential role for PTEN in carcinogenesis and progression. Our findings are in line with literature data [13, 14, 25, 31]. However, our study provides novel insight by reporting on the relative performance of three commonly used antibodies. These novel data should raise caution regarding the interpretation of results obtained using various antibodies, and call for a need to establish standardized and validated protocols to utilize this potentially valuable tool.

We couldn’t find significant difference of staining intensity within tumor regions and matched lymph node metastasis. We only observed a trend of gradual decrease towards the invasive front. This is in contrast to multiple studies claiming rather heterogeneous PTEN expression in CRC based on detection by IHC [18, 27]. Furthermore, PTEN expression was similar in main tumor mass and in lymph node metastasis. These data are in agreement with a finding of strong concordance between PTEN status in primary CRC and corresponding liver metastases [12, 28]. We found no statistically significant differences between the localization of tumor in the large bowel and PTEN expression. In contrast, others found lower PTEN-expression in distal tumors as compared to proximal tumors [25, 34, 35].

In our study, we found no correlations between clinicopathological parameters such as pT, Dukes, clinical stage and PTEN expression levels as determined by the three different antibodies.

Variable results have been reported regarding correlation between PTEN expression and clinicopathological parameters of CRC [13, 15, 36]. Significant correlation of advanced TNM stage with a loss of PTEN expression was referred by Sawai et al. [12]. Taniyama et al. found no correlation between PTEN expression and stage or grade in sporadic CRC [17]. So, there are variable results reported regarding correlations between clinical stage and/or prognosis and PTEN expression levels determined by IHC. Based on our data, we reason that this might be due to a differential performance of various antibodies used for detection, and may be due to a lack of standardization with regard to both the technical performance of staining and analytic techniques.

In our study, KRAS status was correlated with PTEN expression only based on staining with the Neomarkers antibody and there was no correlation found with either the DAKO or the CellSignaling antibody. However, this was detected in those few cases which possessed mutation on exon 13. In this respect the negative findings with the latter two antibodies are in agreement with other studies that did not find statistically significant correlation between PTEN expression and mutations of PIK3CA or KRAS/NRAS/BRAF in primary CRC tissue samples or in their liver metastases [28]. No correlation was found between grade and PTEN status with any antibodies. These findings are similar to what was found by Lin and Jin et al. [37, 38].

The occurrence of MSI in sporadic CRC is 10-15 % [39]. A study identified the presence of frameshift mutations of these poly(A) tracts in colorectal cancers, suggesting that the PTEN gene might be a target of MSI-based colorectal carcinogenesis [40]. In our patient population the occurrence of MSI phenotype was 5.5 % and there was no significant correlation between MSI phenotype and PTEN-expression. However, the low number of MSI tumors in our study renders this comparison underpowered.

When assessing the prognostic role of PTEN expression by dichotomizing CRCs to PTEN-normal and PTEN-loss cases, we found that PTEN-loss determined by any antibodies was not prognostic for relapse-free survival in any setting. Correspondingly, the ROC analysis failed to split the cohort on the basis of PTEN H-score. Bohn et al. found both prognostic and predictive differences between either colonic or rectal localization of PTEN loss: their separate analysis of rectal and colonic cancers revealed a significant association between PTEN status and overall survival in rectal cancers only [41], however, in our analysis rectal tumors were underrepresented. On the other hand, others - in line with our results - did not find any association between PTEN loss and prognosis [28, 37, 38].

## Conclusion

We utilized three different antibodies for the immunohistochemical assessment of PTEN expression in 55 colorectal cancer cases, with an emphasis on identifying locoregional, intratumoral and intracellular staining patterns. Our approach applied three histoscore methods combining the intensity and frequency of PTEN staining. Dako and CellSignaling antibodies had significantly concordant cytoplasmic staining pattern with each other and both detected a decrease in PTEN-expression in all tumor areas compared to normal mucosa, but found no correlation with clinicopathological parameters. IHC staining with the Neomarkers antibody indicated distinctly nuclear pattern and did not justify significant decrease of PTEN-expression in tumor vs. normal. Neomarkers H-scores did not correlate with pT, Dukes, or TNM stage, but did correlate with K-RAS status. Neither applied antibody showed prognostic value in any settings. In our study, the different staining properties of the 3 antibodies made the comparison hard, even if sophisticated evaluation was applied with three different H-scores or a dichotomization for PTEN-loss with different thresholds.

We concluded that the prognostic, predictive potential and clinicopathological relevance of immunohistochemical determination of PTEN status remains uncertain. To overcome this issue more standardized studies relying on previous validation studies (like Sangale et als’) on more numerous and homogenous patients with standardized protocols should be performed possibly involving digital evaluation methods for immunohistochemistry.

## Abbreviations

BEVA, BEVAcizumab; CETUX, CETUXimab; CRC, ColoRectal Cancer; CTX, ChemoTherapy; EGFR, epidermal growth factor receptor; FFPE, formalin-fixed paraffin-embedded; FISH, Fluorescence In Situ Hybridization; IHC, immunohistochemistry; MMR, mismatch repair; MSI, microsatellite instable; MSS, microsatellite stable; NA, not available; PTEN, phosphatase and TENsin homologue

## Additional file

Additional file 1: Figure S1. The respective antibodies detecting PTEN loss. The DAKO, Neomarkers and CellSignaling antibodies showing intact staining in the normal colon mucosa and preserved staining in PTEN normal tumors and lower expression than normal mucosa in carcinomas with PTEN loss. (PDF 1033 kb)
