# Supplementary figures and images for: In depth evaluation of the prognostic and predictive utility of PTEN immunohistochemistry in colorectal carcinomas: performance of three antibodies with emphasis on intracellular and intratumoral heterogeneity
Source: Diagn Pathol. 2016 Jul 8;11:61. doi: 10.1186/s13000-016-0508-0 (PMC4939017; doi:10.1186/s13000-016-0508-0)

|               | Normal mucosa                                                                      | PTEN positive                                                                        | PTEN negative                                                                         |
|---------------|------------------------------------------------------------------------------------|--------------------------------------------------------------------------------------|---------------------------------------------------------------------------------------|
| Dako          | 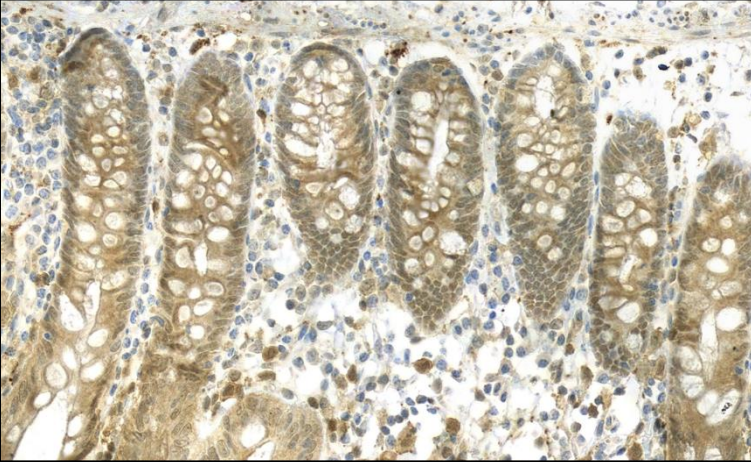    | 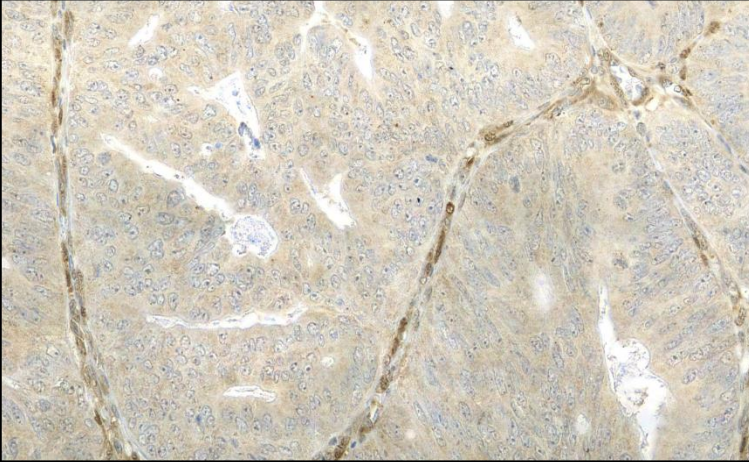    | 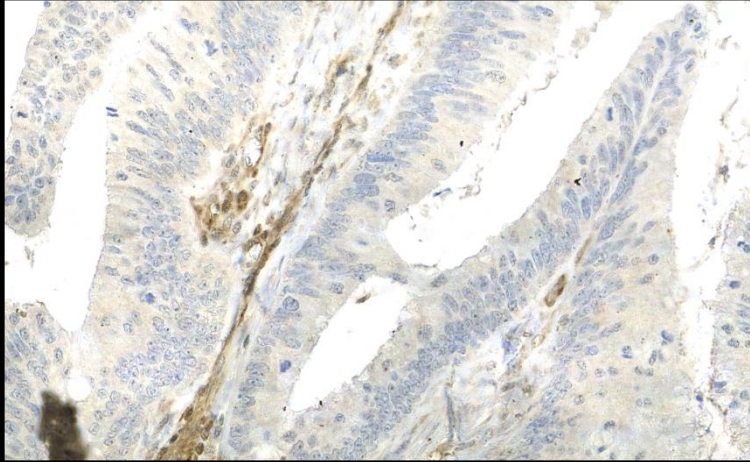    |
| Neomarker     | 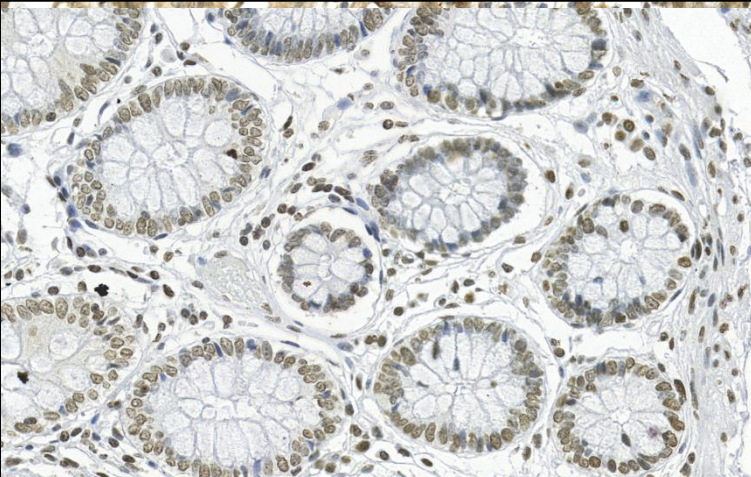  | 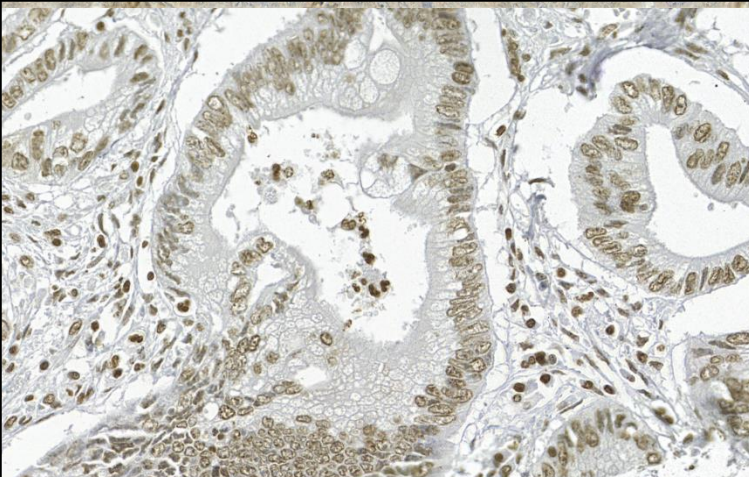  | 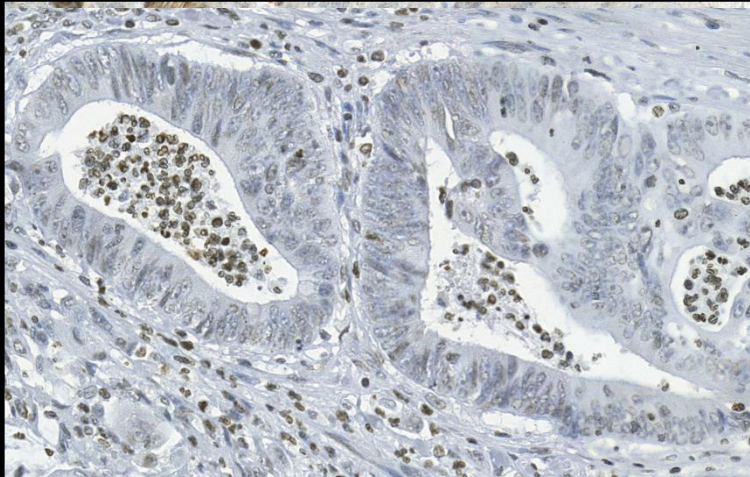  |
| CellSignaling | 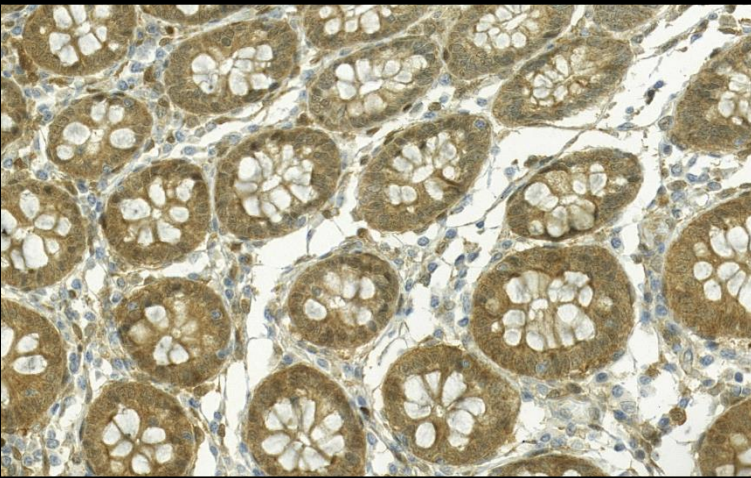 | 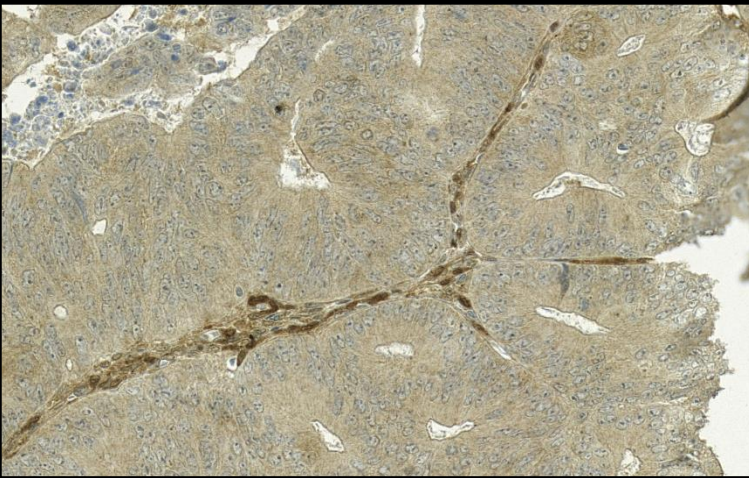 | 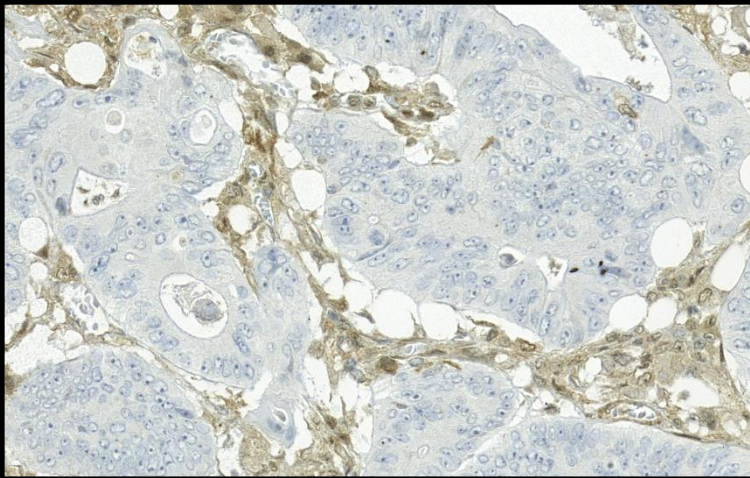 |

Supplement: Additional file 1: Figure S1. — The respective antibodies detecting PTEN loss. The DAKO, Neomarkers and CellSignaling antibodies showing intact staining in the normal colon mucosa and preserved staining in PTEN normal tumors and lower expression than normal mucosa in carcinomas with PTEN loss. (PDF 1033 kb) [file 13000_2016_508_MOESM1_ESM.pdf]
